# Genome Sequences of Anelloviruses, a Genomovirus, Microviruses, Polyomaviruses, and an Unclassified Caudovirus Identified in Vaginal Secretions from South African Adolescents

**DOI:** 10.1128/mra.01143-22

**Published:** 2022-12-19

**Authors:** Adijat O. Jimoh, Christina Balle, Bryan Brown, Colin Feng, Enock Havyarimana, Iyaloo N. Konstantinus, Katherine Gill, Linda-Gail Bekker, Jo-Ann S. Passmore, Heather B. Jaspan, Arvind Varsani, Anna-Ursula Happel

**Affiliations:** a Department of Pathology, Institute of Infectious Disease and Molecular Medicine, University of Cape Town, Cape Town, South Africa; b Institute of Infectious Disease and Molecular Medicine, University of Cape Town, Cape Town, South Africa; c Genetics, Genomics, and Bioinformatics Department, National Biotechnology Development Agency, Abuja, Nigeria; d Seattle Children’s Research Institute, Seattle, Washington, USA; e Department of Pediatrics, University of Washington School of Medicine, Seattle, Washington, USA; f School of Infection and Immunity, University of Glasgow, Glasgow, United Kingdom; g Namibia Institute of Pathology, Windhoek, Namibia; h Desmond Tutu HIV Centre, University of Cape Town, Cape Town, South Africa; i National Health Laboratory Service, Cape Town, South Africa; j NRF-DST Centre of Excellence in HIV Prevention, Centre for the AIDS Programme of Research in South Africa, Durban, South Africa; k Division of Medical Virology, Department of Pathology, University of Cape Town, Cape Town, South Africa; l Department of Global Health, University of Washington, Seattle, Washington, USA; m The Biodesign Center for Fundamental and Applied Microbiomics, Center for Evolution and Medicine and School of Life Sciences, Arizona State University, Tempe, Arizona, USA; n Structural Biology Research Unit, Department of Integrative Biomedical Sciences, University of Cape Town, Cape Town, South Africa; DOE Joint Genome Institute

## Abstract

Other than for papillomaviruses, there is a paucity of whole-genome sequences for bacteriophages and eukaryote-infecting viruses isolated from the female genital tract. Here, we report the genome sequences of 16 microviruses, 3 anelloviruses, 2 polyomaviruses, 1 genomovirus, and 1 caudovirus that were identified in vaginal secretion samples from adolescents in South Africa.

## ANNOUNCEMENT

**FIG 1:**
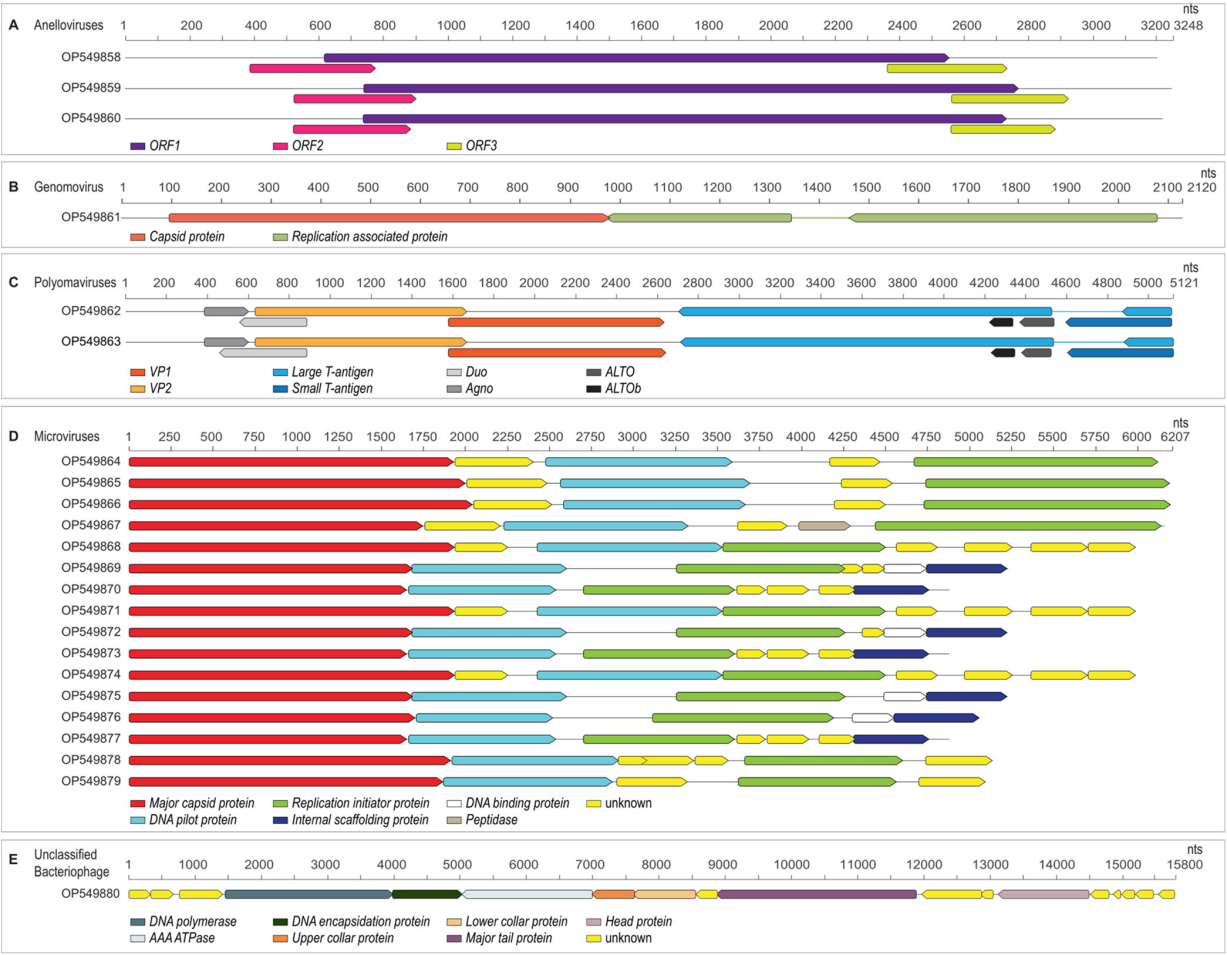
Linearized representations of the genome organization of the anelloviruses (A), genomovirus (B), polyomaviruses (C), microviruses (D), and unclassified caudovirus (E) identified in this study.

Although viruses are frequently present in the female genital tract of asymptomatic women (1), few complete nonpapillomavirus genome sequences are publicly available, thus limiting our ability to conduct extensive vaginal virome studies. Forty-three vaginal lateral wall swab samples were collected from 15- to 19-year-old HIV-seronegative adolescent female participants enrolled between 2015 and 2017 in a randomized trial of hormonal contraceptives in Cape Town, South Africa (ClinicalTrials registration number NCT02404038) (2). Swab samples were suspended in phosphate-buffered saline (PBS) and filtered through 0.45-μm syringe filters, and viral DNA was extracted using the High Pure viral nucleic acid extraction kit (Roche Life Sciences, USA). Viral DNA was enriched for circular DNA molecules by using rolling circle amplification (RCA) with the Illustra TempliPhi kit (GE HealthCare, USA). Library preparation and quantitation were performed by the University of Washington Northwest Genomics Center using the KAPA HyperPrep kit without amplification (KR0961 v1.14) protocols. Forty-three libraries were generated from the RCA products and sequenced in multiplex mode in a single run on a NovaSeq 6000 system. All bioinformatic tools were run with default settings. Raw reads were trimmed using Trimmomatic v0.39 (3) and then *de novo* assembled with metaSPAdes v3.12.0 (4). Virus-like contigs in the assembled data set were identified using BLASTx (5) with a RefSeq virus protein database (RefSeq release 207). Complete genomes were determined based on terminal repeats using a custom script. In 10 samples, we identified complete genome sequences of microviruses (*n* = 16), anelloviruses (*n* = 3), polyomaviruses (*n* = 2), a genomovirus (*n* = 1), and a caudovirus (*n* = 1). Reads were mapped to the viral contigs using BBMap v38.94 (6), showing read depths of 3.4× to 640.53×, with 100 to 22,016 mapped reads per genome (Table 1). Genome sequences and their open reading frames (ORFs) were visualized using Geneious Prime software v2022.2 (Biomatters Ltd., New Zealand). The genome organization, length, GC content, and read depth for all identified genomes are summarized in Fig. 1 and Table 1.

**TABLE 1:** Summary of the viruses identified in this study and their top BLASTn hits

Genus	Species	GenBank accession no.	Virus	Sample identification no.	SRA accession no.	Read depth (×)	No. of reads	Length (bp)	GC content (%)	GenBank accession no. for best BLASTn hit^*a*^	Virus for best BLASTn hit	BLAST coverage (%)	*E* value	Identity vs best BLAST hit (%)
*Gammatorquevirus*	Unclassified	OP549858	Torque teno midi virus UC008V3_C344	UC_008V3	SRR21604215	6.7763	142	3,156	42.30	MN776341	Torque teno midi virus SAfiA-567-8	100	0	98.00
*Gammatorquevirus*	Unclassified	OP549859	Torque teno midi virus UC145V1_C197	UC_145V1	SRR21604186	11.2522	244	3,248	43.10	MN776305	Torque teno midi virus SAfiA-866-496	88	0	96.65
*Gammatorquevirus*	Unclassified	OP549860	Torque teno midi virus UC156V1_C016	UC_156V1	SRR21604185	5.4398	117	3,213	41.70	MN779219	Torque teno midi virus SAfiA-666-25	83	0	96.63
*Gemykrogvirus*	Gemykrogvirus sewopo1	OP549861	Genomovirus UC145V1_C621	UC_145V1	SRR21604186	7.0467	100	2,120	49.00	KJ547634	Sewage-associated gemycircularvirus 4	100	0	98.44
*Betapolyomavirus*	*Human polyomavirus 2*	OP549862	JC polyomavirus UC156V1_C027	UC_156V1	SRR21604185	223.5409	7,602	5,112	40.50	MF662198	JC polyomavirus strain #16	100	0	99.74
*Betapolyomavirus*	*Human polyomavirus 2*	OP549863	JC polyomavirus UC167V3_C026	UC_167V3	SRR21604179	12.1984	415	5,121	40.20	AB127017	JC polyomavirus SO-6	100	0	99.98
Unclassified	Unclassified	OP549864	Microvirus UC029V2_C004	UC_029V2	SRR21604176	531.0064	21,575	6,126	32.10	BK058019	*Microviridae* sp. isolate ctYpv30	8	2.00E−61	73.19
Unclassified	Unclassified	OP549865	Microvirus UC072V2_C071	UC_072V2	SRR21604206	28.8202	1,166	6,190	32.60	BK030845	*Microviridae* sp. isolate ctEKz11	8	4.00E−58	75.28
Unclassified	Unclassified	OP549866	Microvirus UC094V2_C111	UC_094V2	SRR21604199	234.8924	9,672	6,207	35.50	BK058019	*Microviridae* sp. isolate ctYpv30	6	1.00E−27	73.74
Unclassified	Unclassified	OP549867	Microvirus UC141V2_C001	UC_141V2	SRR21604187	8.9501	367	6,155	40.00	BK053425	*Microviridae* sp. isolate ctrXY8	45	0	89.82
Unclassified	Unclassified	OP549868	Microvirus UC145V1_C17	UC_145V1	SRR21604186	10.3316	402	5,990	38.30	BK050733	*Microviridae* sp. isolate ctrRR16	90	0	87.31
Unclassified	Unclassified	OP549869	Microvirus UC145V1_C34	UC_145V1	SRR21604186	17.115	549	5,216	45.90	KT264760	Gokushovirus WZ-2015a 12Fra21	95	0	90.70
Unclassified	Unclassified	OP549870	Microvirus UC145V1_C49	UC_145V1	SRR21604186	164.8787	5,322	4,888	44.60	BK028328	*Microviridae* sp. isolate ctVzM3	93	0	84.01
Unclassified	Unclassified	OP549871	Microvirus UC146V1_C93	UC_146V1	SRR21604183	3.0868	123	5,990	38.30	BK050733	*Microviridae* sp. isolate ctrRR16	90	0	87.31
Unclassified	Unclassified	OP549872	Microvirus UC146V1_C112	UC_146V1	SRR21604183	5.5169	192	5,216	45.90	KT264760	Gokushovirus WZ-2015a 12Fra21	95	0	90.70
Unclassified	Unclassified	OP549873	Microvirus UC146V1_C125	UC_146V1	SRR21604183	53.0215	1,731	4,888	44.60	BK028328	*Microviridae* sp. isolate ctVzM3	93	0	84.01
Unclassified	Unclassified	OP549874	Microvirus UC156V1_C009	UC_156V1	SRR21604185	3.4043	136	5,990	38.30	BK050733	*Microviridae* sp. isolate ctRR16	90	0	87.31
Unclassified	Unclassified	OP549875	Microvirus UC156V1_C022	UC_156V1	SRR21604185	6.2874	219	5,216	45.90	KT264760	Gokushovirus WZ-2015a 12Fra21	95	0	90.70
Unclassified	Unclassified	OP549876	Microvirus UC156V1_C034	UC_156V1	SRR21604185	7.0739	218	5,050	46.00	BK033216	*Microviridae* sp. isolate ct9Vh3	92	0	89.91
Unclassified	Unclassified	OP549877	Microvirus UC156V1_C041	UC_156V1	SRR21604185	56.7246	1,851	4,888	44.60	BK028328	*Microviridae* sp. isolate ctVzM3	93	0	84.01
Unclassified	Unclassified	OP549878	Microvirus UC156V1_C024	UC_156V1	SRR21604185	640.5251	22,016	5,138	40.80	BK046696	*Microviridae* sp. isolate ctqGD2	85	0	88.39
Unclassified	Unclassified	OP549879	Microvirus UC156V1_C026	UC_156V1	SRR21604185	92.011	3,139	5,098	40.20	BK046696	*Microviridae* sp. isolate ctqGD2	81	0	85.99
Unclassified	Unclassified	OP549880	Bacteriophage UC074V2_C064	UC_074V2	SRR21604203	44.2191	4,550	15,800	37.20	BK039135	Bacteriophage isolate ctVgq3	98	0	90.48

a Based on BLAST+ v2.13.0 (https://blast.ncbi.nlm.nih.gov/Blast.cgi).

Anelloviruses are circular, single-stranded DNA (ssDNA), vertebrate-infecting viruses (1.6 to 3.9 kb in size) (7, 8). The three identified anelloviruses (GenBank accession numbers OP549858 to OP549860) shared >95.00% nucleotide identity with previously identified sequences (Table 1) belonging to the genus *Gammatorquevirus* but could not be assigned to any species described previously (9). All three genomes had three overlapping ORFs, characteristic of the *Anelloviridae* family (Fig. 1A). Genomoviruses are circular ssDNA viruses with two major ORFs, encoding an RCA-associated protein and a capsid protein (Fig. 1B) (10–12). The genomovirus (GenBank accession number OP549861) shared 98.44% nucleotide identity with sewage-associated gemycircularvirus 4, which is part of the genus *Gemykrogvirus* and species Gemykrogvirus sewopo1 (13). Polyomaviruses are DNA viruses that infect mammals, birds, fish, and arachnids (4 to 7 kb) (14–18). Polyomaviruses have characteristic major ORF arrangements, including large and small T-antigens and VP1 and VP2 capsid proteins (Fig. 1C) (16). Both polyomaviruses (GenBank accession numbers OP549862 and OP549863) shared >97.00% identity with John Cunningham (JC) polyomaviruses (genus *Betapolyomavirus*, species *Human polyomavirus 2*). Microviruses are ssDNA bacteriophages with circular genomes (4.4 to 6.1 kb) (19) and have been identified in multiple environment types, including the female genital tract (20–28). The 16 microviruses (GenBank accession numbers OP549864 to OP549879) shared ~73.00 to 90.00% pairwise identity with other microviruses, with varying genome coverage (Table 1), but were not assigned to any currently established genus. All 16 genomes encoded a major capsid protein and replication initiation protein. Additional proteins identified included DNA pilot and internal scaffolding proteins (Fig. 1D). The unclassified caudovirus (GenBank accession number OP549880) encoded a major tail protein, with several hypothetical proteins (Fig. 1E), and shared 98.00% identity with bacteriophage isolate ctVgq3, which was previously identified from the vaginal fornix (26).

Ethical approval for the study was obtained from the Human Research Ethics Committee at the University of Cape Town (HREC approval number 801/2014).

### Data availability.

The sequences have been submitted to NCBI under BioProject accession number PRJNA881266, BioSample accession numbers SAMN30889804, SAMN30889810, SAMN30889821, SAMN30889823, SAMN30889827, SAMN30889838, SAMN30889839, SAMN30889840, SAMN30889842, and SAMN30889845, SRA accession numbers SRR21604176, SRR21604179, SRR21604183, SRR21604185, SRR21604186, SRR21604187, SRR21604199, SRR21604203, SRR21604206, and SRR21604215, and GenBank accession numbers OP549858, OP549859, OP549860, OP549861, OP549862, OP549863, OP549864, OP549865, OP549866, OP549867, OP549868, OP549869, OP549870, OP549871, OP549872, OP549873, OP549874, OP549875, OP549876, OP549877, OP549878, OP549879, and OP549880.
